# Prevalence, Demographic, and Clinical Correlates of Likely PTSD in Subscribers of Text4Hope during the COVID-19 Pandemic

**DOI:** 10.3390/ijerph18126227

**Published:** 2021-06-09

**Authors:** Reham Shalaby, Medard K. Adu, Taelina Andreychuk, Ejemai Eboreime, April Gusnowski, Wesley Vuong, Shireen Surood, Andrew J. Greenshaw, Vincent I. O. Agyapong

**Affiliations:** 1 Department of Psychiatry, Faculty of Medicine and Dentistry, University of Alberta, Edmonton, AB T6G 2B7, Canada; rshalaby@ualberta.ca (R.S.); medard@ualberta.ca (M.K.A.); eboreime@ualberta.ca (E.E.); andy.greenshaw@ualberta.ca (A.J.G.); 2 Department of Psychiatry, Cumming School of Medicine, University of Calgary, Calgary, AB T2N 1N4, Canada; taelina.andreychuk@ucalgary.ca; 3 Alberta Health Services, Addiction & Mental Health, Edmonton, AB T5K 2J5, Canada; April.Gusnowski@albertahealthservices.ca (A.G.); Wesley.Vuong@albertahealthservices.ca (W.V.); shireen.surood@albertahealthservices.ca (S.S.)

**Keywords:** PTSD, Text4Hope, family support, COVID-19, depression, stress, anxiety

## Abstract

Background: During the COVID-19 pandemic, people may experience increased risk of adverse mental health, particularly post-traumatic stress disorder (PTSD). Methods: A survey measured stress, anxiety, depression, and PTSD symptoms in Text4Hope subscribers using the Perceived Stress Scale, Generalized Anxiety Disorder 7-Item Scale, Patient Health Questionnaire-9, and PTSD Checklist for DSM-5 Part 3, respectively. A Chi-square test and multivariate logistic regression were employed. Results: Most respondents were 41–60 years old (49.5%), Caucasian (83.3%), with post-secondary education (92.1%), employed (70.3%), married/cohabiting/partnered (64.9%), and homeowners (71.7%). Likely PTSD was reported in 46.8% of the respondents. Those who were afraid to contract the coronavirus had a history of depression before the pandemic, and those who received counselling during the pandemic exhibited a high prevalence of likely PTSD (OR (1.7 to 2.2)). Significant lower odds of likely PTSD were observed among subscribers who received absolute support from family/friends. Conclusions: This paper presents findings on the prevalence of likely PTSD and identified vulnerable groups during the COVID-19 pandemic. Our results support the proposal that public health advice should incorporate mental health wellness campaigns aiming to reduce the psychological impact of pandemics.

## 1. Introduction

Post-traumatic stress disorder (PTSD) is defined as “the development of symptoms related to intrusion, avoidance, negative alterations in cognitions, mood, and arousal and reactivity following exposure to a traumatic event” [1]. Such events may include natural disasters, a serious traffic accident, terrorist act, conflict, or sexual assault, among others [2]. The defining attribute of a traumatic incident is its ability to elicit fear, helplessness, or horror in response to the threat of possible injury or death [3]. Thus, a patient must have experienced such event(s) and presented with symptoms such as reliving the event and avoiding stimulus reminders (triggers) of the event for about four weeks, to be diagnosed with PTSD [1].

Universal population studies indicate that 28% to 90% of people in high-income countries have been exposed to at least one traumatic event in their course of life; the most frequent events are unanticipated bereavement, road traffic accidents, and physical assault [4,5]. Despite this high exposure to stressors, the prevalence of PTSD ranges from 1.3% to 8.8% [6]. PTSD can occur in people of any ethnicity, nationality, or culture, and at any age. PTSD affects approximately 3.5% of U.S. adults every year, and an estimated 1 in 11 people will be diagnosed with PTSD in their lifetime; women are twice as likely as men to have PTSD [1]. According to the Government of Canada, three in four Canadians are at risk of exposure to one or more events throughout their lifetime that may lead to PTSD [7]. The latest estimated prevalence of lifetime PTSD is 9.2%, while the current prevalence (past month) was estimated at 1.7%, with an expectant higher risk reported among Indigenous people, refugees, and the LGBTQ2S+ community [7,8].

The outbreak of the Coronavirus Disease-19 (COVID-19), which was first identified in Wuhan (China) in December 2019, was declared to be a global pandemic that constitutes a public health emergency of international concern by the World Health Organization (WHO) in early 2020 [9]. There were about 77.9 million documented infections and 1.79 million deaths worldwide as of 22 December 2020 [10,11]. The COVID-19 pandemic presents some characteristic features that increase vulnerability to mental health problems including PTSD. It has been shown that PTSD could result from the news of unprecedented numbers of seriously ill patients, the uncertainty of the course of the disease, high mortality rates, and the absence of a definitive treatment [10,12]. The experiences faced by severely ill COVID-19 patients in the form of symptoms of extreme stressors, including fear of eminent death from the threatening illness, the feeling of loss of control, and the pain associated with medical interventions such as endotracheal intubation [13], constitute the first diagnostic criterion for PTSD [14,15].

Pandemic-associated psychological trauma and related PTSD are not limited to survivors of COVID-19. Frontline healthcare staff and relatives who had a family member die as a result of COVID 19 may also have developed some form of PTSD from witnessing other traumatic events, as was reported with previous coronavirus epidemics, such as the Middle East respiratory syndrome (MERS) and the severe acute respiratory syndrome (SARS) [16,17]. With respect to these epidemics, close to 42% of survivors developed PTSD after a year, with similar population figures remaining above the cut-off up to four years post-pandemic [17].

The factors impacting PTSD are quite diverse, including sociodemographic factors and the pre-existing mental health conditions, among others. Being a female or of a relatively younger age were both perceived as a significant risk for developing PTSD, particularly when comorbid with depression [13]. Similarly, among the different ethnicity groups, minorities (non-white) people in the USA were described to be at more at risk to experience PTSD, with less of a likelihood to seek treatment support, either by visiting doctors, counsellors, or going to hospitals, compared to white people [5]. Likewise, pre-morbid depression and anxiety have been linked to a higher conditional risk of PTSD [5].

Given this precedent, the anticipation of PTSD in the more widespread COVID-19 pandemic necessitates monitoring and management of this expected negative impact [10,12,18].

This study aimed to evaluate the prevalence, demographic, and clinical correlates of likely PTSD in subscribers of “Text4Hope,” an intervention developed at the peak of the first wave of the pandemic to reduce the psychological treatment gap and mitigate anxiety and stress related to the COVID-19 crisis among Canadians [19]. Collateral effects of the pandemic include mental health impacts in terms of anxiety, fear, hopelessness, and stigmatization, which additionally may hinder access to medical and mental health interventions [18]. The Text4Hope program broadcasted daily supportive text messages to the mobile phones of Canadians who subscribed to the service, thereby expanding access to mental health support and offering this service even during self-isolation and quarantine. 

## 2. Materials and Methods

### 2.1. Study Design and Ethics Approval

This study comprised of a cross-sectional survey. Categorical data on sociodemographic and clinical variables were collected through an online survey. To enable blind review by the study team members, some information was masked for that process. Institutional ethics approval was provided for this study by the University of Alberta Health Research Ethics Board (approval PRO00086163).

### 2.2. Participant Recruitment and Data Collection

A self-administered questionnaire was administered to Text4Hope subscribers between 18 June and 19 August 2020, after three months of service use. Text4Hope is a mobile-based texting program introduced by Alberta Health Services (AHS) in partnership with other health organizations to provide Albertans with mental health support during the COVID-19 pandemic [20]. Self-subscription to the program occurred by texting “COVID19Hope” to a short code number to receive free daily supportive text messages over a three-month period. Messages were crafted on the basis of cognitive behavioral therapy (CBT) principles by AHS psychiatrists and mental health therapists, including the authors of the study (VA, MH). Survey questions were programmed into Select Survey, an online survey tool. All Text4Hope subscribers who completed the three-month program were invited to complete the survey, which included demographic and clinical questions including gender, age, ethnicity, highest level of education completed, employment, relationship and housing status, history of mental illness, and use of psychotropic medication before the pandemic. 

### 2.3. Outcomes and Measures

The survey measured PTSD symptoms in subscribers using the PTSD Checklist for DSM-5 (PCL-5) Part 3 [21]. PCL-5 is a psychometrically sound instrument and consists of 20 questions, and the respondents’ scores range from 0 to 80. The scale demonstrated good internal consistency (alpha = 0.96), test-retest reliability (r = 0.84), and convergent and discriminant validity [22]. 

The survey additionally included questions related to exposure to COVID-19 pandemic news, fears of contracting the coronavirus infection, and whether the subscriber had a family member or friend test positive for coronavirus infection. Subscribers were also asked about the levels of support they received from family and friends, their employer, and the Government of Canada during the pandemic.

No incentives were offered and completing the survey was voluntary and was not a prerequisite for access to Text4Hope. With 36,176 active subscribers receiving the exit survey link, a sample size of 1037 survey respondents was needed to estimate the prevalence of PTSD likelihood during the COVID-19 pandemic with a confidence level of 95% and a 3% margin of error.

### 2.4. Statistical Analysis 

Results were analyzed using SPSS Version 20 [23]. Descriptive statistics were provided for demographic, clinical, and other variables based on gender analysis. Cross-tabular analyses using the Chi-square test explored relationships, categorical predictors, and the likelihood that respondents self-reported PTSD symptoms during the COVID-19 pandemic. Based on factors previously examined [5,13], we were interested in examining the different factors that may ultimately lead to the outcome of likely PTSD. Two categories were calculated based on the PCL-5 total score: (0–32) for not likely PTSD and (33–80) for more likely PTSD. 

Variables with a statistically significant or near significant relationship (*p* ≤ 0.1) to the likelihood of respondents to self-report PTSD (PCL-5 score of 33 or more for likely PTSD) were included in a logistic regression model. Correlational analysis was performed before running the regression analysis to exclude any strong intercorrelations (Spearman’s correlation coefficient of 0.7 to 1.0 or −0.7 to −1.0) among predictor variables. Odds ratios (OR) and confidence intervals from the binary logistic regression analysis were examined to determine predictor variables for respondents to self-report PTSD symptoms during the COVID-19 pandemic, controlling for the other variables. There was no imputation for missing data and the data analyzed and reported reflect the number of responses for each question. 

## 3. Results 

Of 36,176 subscribers, 1079 respondents completed the exit survey giving a response rate of 3.0%. In all, 96 (8.9%) of subscribers identified as male, 953 (88.3%) identified as female, and 11 (1.0%) identified as other gender. Table 1 and Table 2 provide descriptive measures of demographic and clinical characteristics of the respondents by gender. From Table 1, most respondents were in the age group of 41–60 years (49.5%), identified as Caucasian (83.3%), had post-secondary education (92.1%), were employed (70.3%), were married, cohabiting, or partnered (64.9%), and were homeowners (71.7%). Regarding COVID-19-related variables, the majority reported that they listened daily to COVID-19 pandemic news updates (64.9%), watched daily the images of COVID-19-related deaths/sickness (34.1%), did not lose employment due to COVID-19 (67.0%), received absolute support from family/friends (49.9%), received absolute support from employers (39.3%), received absolute support from the Government of Canada (28.2%), and sought and received mental health counselling during the pandemic (73.7% and 69.7%, respectively).

Table 2 indicates that just over half of the respondents reported having no history of any mental health disorder (51.3%), while almost a third reported having a history of either anxiety or depressive disorder (31.2% and 30.8%, respectively), with the highest prevalence observed among other gender for the two conditions. Respondents who reported receiving antidepressant medications before the pandemic represented the highest proportion (28.2%), compared to respondents who reported use of other psychotropic medications (<10%). Again, other gender had the highest rates of receiving all psychotropic medications except for mood stabilizers, where males reported the highest intake rate (15.6%).

Self-isolation or self-quarantine was reported by around 1 in 4 respondents (26.4%), and around 1 in 12 (7.9%) reported having a family member or friend test positive for coronavirus. More than 8 in 10 respondents were afraid of being infected (83.2%). Finally, almost a half of the respondents scored positive for the likelihood of PTSD based on the PCL-5 scale (46.8%), with other gender reporting the highest prevalence (66.7%).

### 3.1. Univariate Analysis

Table 3 summarizes relationships between demographic and clinical antecedents and likely PTSD: 23 out of 28 predictor variables were significantly or near significantly related to likely PTSD (*p* ≤ 0.1). Furthermore, 2 out of the 23 variables did not proceed to the regression model as they showed a high correlation with other variables (‘no history of mental health diagnosis before the pandemic’ and ‘on no psychotropic medication before the pandemic’).

### 3.2. Logistic Regression

The multivariate model including all 21 variables was statistically significant; Χ2 (42, N = 760) = 282.53, *p* < 0.001, and it correctly classified 74.6% of cases, indicating that the model could distinguish between respondents who did or did not exhibit likely PTSD during the COVID-19 pandemic. The model accounted for 31.0% (Cox and Snell R 2) to 41.4% (Nagelkerke R 2) of the variance in the likelihood of the respondents to present with PTSD. The goodness-of-fit statistic of the logistic regression model was assessed using Hosmer-Lemeshow goodness-of-fit test, which revealed there was not enough evidence to say that the model was a poor fit (3.13, *p* = 0.93).

Table 4 shows the results of the multivariate logistic regression analysis. In summary, the following groups indicated significant higher odds of experiencing PTSD: those who were afraid to contract the coronavirus, respondents who had a history of depression before the pandemic, and those who received counselling during the pandemic, with around a two times greater likelihood of reporting PTSD during the COVID-19 pandemic for each variable compared to respondents in the other categories of their respective variables (OR ranges from 1.70 to 2.20). Subscribers who received absolute support from family/friends had lower odds of reporting PTSD during the pandemic compared to those who did not. Respondents who reported receiving only limited support from their employer were twice as likely to achieve criteria for PTSD, compared to respondents who received absolute support from their employer (OR = 2.02, 95% CI: 1.06–3.83). In addition, Indigenous people were about four times as likely to achieve criteria for PTSD compared to those who identified as Caucasian (OR = 3.90; 95% CI: 1.10–13.78). Similarly, subscribers who reported renting had 67% higher odds of achieving the criteria for PTSD compared to those who owned homes (OR = 1.67; 95% CI: 1.01–2.78).

## 4. Discussion

The results of this study indicate that almost 50% of subscribers reported having likely PTSD. After adjusting for confounders, identifying as Indigenous and living in rented accommodations were significantly associated with likely PTSD during the COVID-19 pandemic. Further, the significant correlates of increased odds of experiencing likely PTSD included fear of COVID-19, a history of depression, and a history of receiving counselling. Conversely, our findings suggested that support from family may offer protection against PTSD. A relatively high prevalence of PTSD is not unexpected during stressful periods, where it can rise up to 40% among survivors in the first year after a disaster [7]. A general population-based study conducted to determine the level of COVID-19-related traumatic distress in the Republic of Ireland reported that 17.67% of the population met diagnostic requirements for PTSD [24]. Similarly, a parallel survey conducted in the United Kingdom estimated a 16.79% prevalence of PTSD [25]. Our current study prevalence estimate of 46.8% is high compared with these surveys done in Ireland and the UK. This large divergence may reflect differences in the respective study populations. Text4Hope subscribers, though drawn from the general population, may not represent general population demographics, given that subscribers to Text4Hope may have already been seeking mental health care compared to the respondents in the UK and Ireland studies. Further, the different instruments used in measuring the outcome may also contribute to the observed variance; our study used the PTSD Checklist for DSM-5 (PCL-5) Part 3, while the European studies applied the International Trauma Questionnaire, a self-report measure of ICD-11 PTSD. 

The high odds of experiencing PTSD symptoms were found among respondents in this study who were afraid to contact the coronavirus, had a history of depression, or who received counselling, resonate with other studies in the literature. A case-control study in China reported that more than one-third of patients with a psychiatric diagnosis met diagnostic criteria for PTSD during the COVID-19 pandemic [26]. Similar results were reported up to four years after the SARS-CoV-1 pandemic [27]. Likewise, a cross-sectional study of PTSD symptoms among healthcare workers and public service providers in Norway concluded that participants who had a pre-existing psychiatric diagnosis, higher levels of anxiety, and depression symptoms were associated with more PTSD symptoms [28].

In our study, a significant effect of family support during COVID-19 was strongly associated with a smaller likelihood of PTSD symptoms. This is in accord with a similar result from previous research that examined probable PTSD predictors among survivors of Fort McMurray wildfire six months after the disaster [29], and a Norwegian study that reported emotional support to be weakly protective against PTSD [28]. These findings are consistent with what we understand of the role of support from family and friends of trauma survivors, positively influencing the form of post-traumatic cognition, which is a driver of PTSD symptoms, therefore reducing the likelihood of PTSD [30]. 

Our findings indicate an increased likelihood of PTSD symptoms among respondents who reported self-isolation and/or quarantine during the COVID-19 pandemic, compared to those who did not (52% vs. 45%); however, this difference was not significantly related to expressed PTSD symptoms. This observation is not clearly consistent with the evidence for an association between quarantine experience during epidemics and diverse mental health disorders, including PTSD symptoms [31,32,33]. 

Based on our analysis, Indigenous ethnicity and living in rented accommodations were sociodemographic correlates of having likely PTSD during the pandemic. Housing challenges have been identified as stressors associated with PTSD in previous studies in Canada [34]. These challenges may have been compounded during the pandemic. A review of studies suggests that pandemic-related worries and stressors (e.g., worry of being infected, housing problems, social isolation, and lack of support) may contribute to an increased risk of PTSD [35]. That review also indicates a disproportionately high risk for socio-economically disadvantaged and racialized populations. 

In contrast with our results, other studies highlighted the effect of the female sex along with being married or cohabiting as potential predictors for the development of mental health symptoms during the current pandemic [32,33]. This contrast could be due to the differences in the other variables included in the regression models between studies. 

Overall, according to a recent systematic review and meta-analysis, COVID-19 has threatened the mental health of nearly one-third of the general population, in relation to challenges that include depression, anxiety, and stress [33], which may increase likelihood for the subsequent development of PTSD symptoms. Our study results, coupled with data from similar studies around the world, highlight the need for focused mental health support for vulnerable, minority, socio-economically disadvantaged, and racialized groups during the COVID-19 pandemic. 

Our study is not without limitations, which include the use of self-reported questionnaires, including the PTSD checklist to score those likely to have PTSD, rather than a formal clinician-rated assessment. The use of well-validated and standardized scales, however, mitigates the risk of information bias with self-report questionnaires. Another limitation is selection bias, where our respondents were Text4Hope subscribers who might have opted to the service seeking mental health support, and, therefore, affected the strength of the generalizability of our findings. In addition, this survey is unable to capture the direct effects of COVID-19 among persons with a confirmed diagnosis of PTSD, and this is an interesting area for future investigation. Another limitation is that, unlike stress, anxiety, and depression symptoms, we did not collect the baseline level of PTSD symptoms in our subscribers. As this study focused on uncontrolled real-world events (COVID-19), it was not possible to include a conventional set of controls such as those embodied in a control group design. We were also unable to report the changes in PTSD prevalence from baseline at this time point and we hoped that the six-month follow-up survey, which included the measurement of PTSD symptoms, would shed some light on range and severity of symptoms experienced between the three- and six-month time points. The variables in this study explained only 31%–41.4% of the variance for PTSD likelihood among subscribers. This may necessitate further research exploring additional potential predictors (e.g., childhood adverse experience, previous trauma as adults, and prior diagnosis of PTSD) that may enrich the explanatory value of the regression model.

It is notable that subscribers to the Text4Hope service reported significant improvement in stress and anxiety levels after six weeks [36], and improvements to stress, anxiety, and depression levels after three months of receiving the daily supportive text messages. This indicates that the likely PTSD prevalence rates in subscribers were probably much higher at baseline than the level reported in this three-month survey. Ordinarily, a population-based random sample would have been ideal for this study, but the uncertainties of the pandemic precluded that approach, and Text4Hope subscription was ostensibly randomly subscribed to. We do acknowledge selection bias in the advertisement and recruitment process leading to a likely non-representative sample of the Albertan population. Finally, the study sample is not representative of age or gender for Alberta. As such, the results may not be generalizable and should be interpreted with caution. Given that males made up a fraction of the sample population, the differences we observed must be interpreted with caution.

Notwithstanding these limitations, our study identified potential factors that increase the likelihood for individuals to develop PTSD symptoms during the COVID-19 pandemic. To our knowledge, this is the first study to evaluate likely PTSD and its correlates in Canada during this pandemic.

## 5. Conclusions

The current findings reveal significant factors that have policy implications for the management of the ongoing pandemic. The data support the proposal that public health advice during pandemics should incorporate mental health wellness campaigns aiming to reduce the psychological impact of pandemics. There is increasing attention being paid to this need in the media, and our data may serve to provide evidence-based support for such policy development and implementation. Cost-effective population-level interventions, such as supportive text messaging services, which are geographic-location independent, are free to the end user, do not require expensive data plans, and can reach thousands of people simultaneously [36,37,38,39,40,41,42,43,44,45,46], are useful for addressing PTSD and other psychological symptoms, such as anxiety and depression, during the COVID-19 pandemic.

## Figures and Tables

**Table 1:** Demographic characteristics of the study population and support for respondents.

Variables	Male	Female	Other	Overall
	N (%)	N (%)	N (%)	N (%)
**Age (Years)**				
≤25	10 (10.5)	65 (6.9)	1 (9.1)	76 (7.2)
26–40	25 (26.3)	269 (28.5)	7 (63.6)	301 (28.7)
41–60	40 (42.1)	478 (50.6)	2 (18.2)	520 (49.5)
>60	20 (21.1)	132 (14.0)	1 (9.1)	153 (14.6)
**Ethnicity**				
Caucasian	75 (78.1)	797 (84.0)	8 (72.7)	880 (83.3)
Indigenous	5 (5.2)	24 (2.5)	0 (0.0)	29 (2.7)
Asian	6 (6.2)	56 (5.9)	2 (18.2)	64 (6.1)
Other	10 (10.4)	72 (7.6)	1 (9.1)	83 (7.9)
**Education**				
Less than High School Diploma	5 (6.1)	12 (1.5)	0 (0.0)	17 (1.9)
High School Diploma	5 (6.1)	40 (5.0)	1 (11.1)	46 (5.1)
Post-Secondary Education	71 (86.6)	744 (92.7)	8 (88.9)	823 (92.1)
Other Education	1 (1.2)	7 (0.9)	0 (0.0)	8 (0.9)
**Employment status**				
Employed	53 (64.6)	572 (71.1)	5 (55.6)	630 (70.3)
Unemployed	12 (14.6)	96 (11.9)	2 (22.2)	110 (12.3)
Retired	13 (15.9)	86 (10.7)	1 (11.1)	100 (11.2)
Students	4 (4.9)	31 (3.9)	1 (11.1)	36 (4.0)
Other	0 (0.0)	20 (2.5)	0 (0.0)	20 (2.2)
**Relationship status**				
Married/Cohabiting/Partnered	46 (56.1)	530 (66.1)	4 (44.4)	580 (64.9)
Separated/Divorced	10 (12.2)	84 (10.5)	0 (0.0)	94 (10.5)
Widowed	0 (0.0)	21 (2.6)	1 (11.1)	22 (2.5)
Single	24 (29.3)	161 (20.1)	4 (44.4)	189 (21.2)
Other	2 (2.4)	6 (0.7)	0 (0.0)	8 (0.9)
**Housing status**				
Own home	49 (59.8)	583 (73.2)	4 (44.4)	636 (71.7)
Living with family	12 (14.6)	65 (8.2)	1 (11.1)	78 (8.8)
Renting	21 (25.6)	148 (18.6)	4 (44.4)	173 (19.5)
**Listened to COVID-19 pandemic news updates**				
Not at all	2 (2.3)	22 (2.4)	0 (0.0)	24 (2.4)
Less than once a week	2 (2.3)	39 (4.3)	0 (0.0)	41 (4.0)
About once weekly	6 (6.8)	69 (7.5)	1 (9.1)	76 (7.5)
Every other day	20 (22.7)	192 (21.0)	3 (27.3)	215 (21.2)
Daily	58 (65.9)	592 (64.8)	7 (63.6)	657 (64.9)
**Watched images of COVID-19-related**				
**deaths/sickness**				
Not at all	16 (18.2)	160 (17.5)	1 (9.1)	177 (17.5)
Less than once a week	11 (12.5)	162 (17.7)	3 (27.3)	176 (17.4)
About once weekly	9 (10.2)	136 (14.9)	3 (27.3)	148 (14.6)
Every other day	15 (17.0)	151 (16.5)	1 (9.1)	167 (16.5)
Daily	37 (42.0)	305 (33.4)	3 (27.3)	345 (34.1)
**Lost job due to the COVID-19 pandemic**				
No	59 (67.0)	616 (67.4)	4 (36.4)	679 (67.0)
Yes	9 (10.2)	119 (13.0)	4 (36.4)	132 (13.0)
Did not have a job before the pandemic	20 (22.7)	179 (19.6)	3 (27.3)	202 (19.9)
**Received sufficient support from family and friends**				
Yes, absolute support	49 (55.7)	450 (49.2)	6 (54.5)	505 (49.9)
Yes, some support	22 (25.0)	284 (31.1)	3 (27.3)	309 (30.5)
Yes, but only limited support	8 (9.1)	134 (14.7)	1 (9.1)	143 (14.1)
Not at all	9 (10.2)	46 (5.0)	1 (9.1)	56 (5.5)
**Received sufficient support from employer**				
Yes, absolute support	37 (42.0)	357 (39.2)	3 (27.3)	397 (39.3)
Yes, some support	11 (12.5)	168 (18.4)	3 (27.3)	182 (18.0)
Yes, but only limited support	7 (8.0)	92 (10.1)	0 (0.0)	99 (9.8)
Not at all	5 (5.7)	69 (7.6)	1 (9.1)	75 (7.4)
Not Applicable/Not currently employed	28 (31.8)	225 (24.7)	4 (36.4)	257 (25.4)
**Received sufficient support from the Government of Canada**				
Yes, absolute support	23 (26.1)	254 (28.3)	4 (36.4)	281 (28.2)
Yes, some support	18 (20.5)	222 (24.7)	2 (18.2)	242 (24.3)
Yes, but only limited support	12 (13.6)	149 (16.6)	2 (18.2)	163 (16.3)
Not at all	35 (39.8)	273 (30.4)	3 (27.3)	311 (31.2)
**Sought MH counselling during the pandemic**				
No	63 (71.6)	677 (74.2)	5 (45.5)	745 (73.7)
Yes	25 (28.4)	235 (25.8)	6 (54.5)	266 (26.3)
**Received MH counselling during the pandemic**				
No	52 (59.1)	649 (70.9)	6 (69.7)	707 (69.7)
Yes	36 (40.9)	266 (29.1)	5 (45.5)	307 (30.3)

COVID-19: Coronavirus disease 2019; MH: Mental health.

**Table 2:** Psychiatric history and clinical self-report-based characteristics of respondents.

Variables	Male	Female	Other	Overall
	N %	N %	N %	N %
**History of mental health diagnosis before the pandemic**				
Depressive Disorder	27 (28.1)	293 (30.7)	6 (54.5)	326 (30.8)
Bipolar Disorder	5 (5.2)	24 (2.5)	2 (18.2)	31 (2.9)
Anxiety Disorder	31 (32.3)	294 (30.8)	6 (54.5)	331 (31.2)
Alcohol Abuse	1 (1.0)	7 (0.7)	1 (9.1)	9 (0.8)
Drug Abuse	1 (1.0)	5 (0.5)	1 (9.1)	7 (0.7)
Schizophrenia	1 (1.0)	2 (0.2)	0 (0.0)	3 (0.3)
Personality Disorder	4 (4.2)	19 (2.0)	3 (27.3)	26 (2.5)
No mental health diagnosis	49 (51.0)	491 (51.5)	4 (36.4)	544 (51.3)
**On psychotropic medication before the pandemic**				
Antidepressants	22 (22.9)	270 (28.3)	7 (63.6)	299 (28.2)
Antipsychotics	4 (4.2)	17 (1.8)	0 (0.0)	21 (2.0)
Sleeping tablets	10 (10.4)	79 (8.3)	1 (9.1)	90 (8.5)
Mood stabilizers	15 (15.6)	47 (4.9)	1 (9.1)	63 (5.9)
Benzodiazepines	3 (3.1)	22 (2.3)	0 (0.0)	25 (2.4)
On no psychotropic medication	56 (58.3)	565 (59.3)	4 (36.4)	625 (59.0)
**Self-isolated/self-quarantined**				
No	71 (74.0)	701 (73.6)	7 (63.6)	779 (73.6)
Yes	25 (26.0)	251 (26.4)	4 (36.4)	280 (26.4)
**Had a family member or friend contract coronavirus**				
No	78 (88.6%)	844 (92.4%)	10 (90.9)	932 (92.1%)
Yes	10 (11.4%)	69 (7.6%)	1 (9.1%)	80 (7.9%)
**Was afraid to contract the coronavirus**				
No	24 (27.3)	144 (15.8)	2 (18.2)	170 (16.8)
Yes	64 (72.7)	769 (84.2)	9 (81.8)	842 (83.2)
**Respondents had likely PTSD based on PCL-5 scale**	31 (41.9%)	339 (47.0%)	6 (66.7%)	376 (46.8%)

PTSD: post-traumatic stress disorder; PCL-5: PTSD Checklist for DSM-5 (Diagnostic and Statistical Manual of Mental Disorders).

**Table 3:** Chi-Square test of association between the demographic and clinical antecedents and likely PTSD *.

Variables	PTSD Likely		χ2 Square Value	*p*-Value
	N	% **		
**Gender**			*	0.37
Male	31	41.9		
Female	339	47.0		
Other	6	66.7		
**Age (Years)**			28.02	<0.001
≤25	33	70.2		
26–40	121	56.5		
41–60	173	41.7		
>60	45	36.6		
**Ethnicity**			7.31	0.06
Caucasian	314	46.2		
Indigenous	17	73.9		
Asian	20	47.6		
Other	26	42.6		
**Education**			8.5	0.04
Less than High School Diploma	6	54.5		
High School Diploma	28	68.3		
Post-Secondary Education	338	45.9		
Other Education	2	33.3		
**Employment status**			24.07	<0.001
Employed	259	45.8		
Unemployed	62	62.6		
Retired	28	31.1		
Students	19	67.9		
Other	6	46.2		
**Relationship status**			15.41	0.004
Married/Cohabiting/Partnered	222	42.6		
Separated/Divorced	41	51.9		
Widowed	7	38.9		
Single	99	59.3		
Other	4	50.0		
**Housing status**			40.41	<0.001
Own home	233	40.2		
Living with family	40	70.2		
Renting	97	7.2		
**Lost job due to the COVID-19 pandemic**			11.06	0.004
No	233	42.8		
Yes	55	53.4		
Did not have a job before the pandemic	93	56.0		
**Self-isolated/self-quarantined**			3.71	0.05
No	265	44.7		
Yes	116	52.3		
**Had a family member or friend contract coronavirus**			1.65	0.20
No	356	47.4		
Yes	25	39.1		
**Was afraid to contract the coronavirus**			23.57	<0.001
No	36	27.5		
Yes	346	50.0		
**Have listened to COVID-19 pandemic news updates**			1.48	0.83
Not at all	9	47.4		
Less than once a week	17	53.1		
About once weekly	33	52.4		
Every other day	75	46.0		
Daily	248	46.0		
**Watched images of COVID-19-related deaths/sicknesses**			2.86	0.58
Not at all	66	47.8		
Less than once a week	69	48.3		
About once weekly	51	42.1		
Every other day	57	42.9		
Daily	139	49.5		
**History of depressive disorder before the pandemic**			103.41	<0.001
No	189	34.4		
Yes	193	72.3		
**History of anxiety disorder before the pandemic**			96.72	<0.001
No	187	34.6		
Yes	195	70.9		
**History of bipolar disorder before the pandemic**			7.44	0.01
No	363	45.9		
Yes	19	73.1		
**History of schizophrenia before the pandemic**			*	0.60
No	380	46.7		
Yes	2	66.7		
**No history of mental health diagnosis before the pandemic**			117.39	<0.001
No (positive history)	253	67.3		
Yes (negative history)	129	29.3		
**On antidepressants before the pandemic**			60.96	<0.001
No	220	38.1		
Yes	162	68.1		
**On sleeping tablets before the pandemic**			9.0	0.003
No	334	45.1		
Yes	48	63.2		
**On mood stabilizers before the pandemic**			23.94	<0.001
No	340	44.6		
Yes	42	79.2		
**On benzodiazepines before the pandemic**			15.32	<0.001
No	362	45.6		
Yes	20	87.0		
**On antipsychotics before the pandemic**			9.07	0.003
No	366	46.0		
Yes	16	80.0		
**On no psychotropic medication before the pandemic**			66.46	<0.001
No (on psychotropic medication)	199	65.2		
Yes (not on psychotropic medication)	183	35.8		
**Received sufficient support from family and friends**			108.58	<0.001
Yes, absolute support	123	29.9		
Yes, some support	144	56.9		
Yes, but only limited support	87	74.4		
Not at all	28	80.0		
**Received sufficient support from employer**			39.01	<0.001
Yes, absolute support	106	34.1		
Yes, some support	80	54.1		
Yes, but only limited support	50	62.5		
Not at all	36	64.3		
Not currently employed	108	50.0		
**Received sufficient support from the Government of Canada**			28.28	<0.001
Yes, absolute support	76	33.2		
Yes, some support	98	50		
Yes, but only limited support	59	48		
Not at all	144	56.9		
**Received counselling during the pandemic**			65.55	<0.001
No	216	37.6		
Yes	166	68.6		

* Fisher’s exact test; ** percentage of each category in each variable who had likely PTSD.

**Table 4:** Logistic regression predicting likelihood of respondents presenting with PTSD.

Predictor	B	SE	Wald	df	*p*-Value	Odds Ratio	95% CI for Odds Ratio	
							Lower	Upper
**Age (Years)**								
≤25			1.939	3	0.585			
26–40	0.048	0.500	0.009	1	0.923	1.049	0.394	2.794
41–60	−0.255	0.514	0.246	1	0.620	0.775	0.283	2.121
>60	−0.288	0.605	0.227	1	0.634	0.749	0.229	2.454
**Ethnicity**								
Caucasian			5.847	3	0.119			
Indigenous	1.361	0.644	4.462	1	0.035	3.899	1.103	13.783
Asian	−0.356	0.410	0.755	1	0.385	0.700	0.314	1.564
Other	−0.228	0.362	0.397	1	0.528	0.796	0.391	1.618
**Education**								
Less than High School Diploma			3.958	3	0.266			
High School Diploma	1.469	1.076	1.863	1	0.172	4.346	0.527	35.825
Post-Secondary Education	0.801	1.006	0.634	1	0.426	2.227	0.310	15.990
Other Education	−0.192	1.534	0.016	1	0.901	0.826	0.041	16.680
**Employment status**								
Employed			1.630	4	0.803			
Unemployed	−0.067	0.375	0.032	1	0.859	0.935	0.448	1.952
Retired	−0.262	0.439	0.357	1	0.550	0.770	0.326	1.818
Students	0.643	0.621	1.072	1	0.300	1.902	0.563	6.421
Other	0.002	0.763	0.000	1	0.998	1.002	0.225	4.467
**Relationship status**								
Married/Cohabiting/Partnered			0.549	4	0.969			
Separated/Divorced	−0.119	0.326	0.133	1	0.715	0.888	0.468	1.683
Widowed	0.248	0.668	0.138	1	0.710	1.282	0.346	4.747
Single	0.103	0.244	0.179	1	0.672	1.109	0.687	1.789
Other	0.011	1.002	0.000	1	0.991	1.011	0.142	7.214
**Housing status**								
Own home			4.545	2	0.103			
Living with family	0.602	0.440	1.871	1	0.171	1.825	0.771	4.323
Renting	0.514	0.259	3.923	1	0.048	1.672	1.005	2.780
**Lost job due to the COVID-19 pandemic**								
No			3.509	2	0.173			
Yes	0.443	0.340	1.701	1	0.192	1.557	0.800	3.031
Did not have a job before the pandemic	0.704	0.428	2.709	1	0.100	2.021	0.874	4.673
**Self-isolated/self-quarantined**								
No Yes	0.070	0.209	0.112	1	0.738	1.072	0.712	1.614
**Were afraid to contract the coronavirus**								
No Yes	0.808	0.273	8.767	1	0.003	2.243	1.314	3.830
**Respondents had a history of depression before the pandemic**								
No Yes	0.797	0.279	8.155	1	0.004	2.218	1.284	3.831
**History of anxiety disorder before the pandemic**								
No Yes	0.471	0.246	3.653	1	0.056	1.602	0.988	2.596
**History of bipolar disorder before the pandemic**								
No Yes	−0.043	0.667	0.004	1	0.949	0.958	0.259	3.541
**On antidepressants before the pandemic**								
No Yes	0.313	0.277	1.274	1	0.259	1.367	0.794	2.355
**On sleeping tablets before the pandemic**								
No Yes	−0.034	0.354	0.009	1	0.924	0.967	0.483	1.934
**On benzodiazepine tablets before the pandemic**								
No Yes	0.555	0.857	0.421	1	0.517	1.743	0.325	9.339
**On mood stabilizers before the pandemic**								
No Yes	0.812	0.479	2.873	1	0.090	2.251	0.881	5.754
**On an antipsychotic before the pandemic**								
No Yes	0.249	0.761	0.107	1	0.744	1.282	0.289	5.696
**Received sufficient support from family and friends**								
Yes, absolute support			36.395	3	0.000			
Yes, some support	0.873	0.209	17.463	1	0.000	2.394	1.590	3.604
Yes, but only limited support	1.554	0.302	26.391	1	0.000	4.730	2.615	8.558
Not at all	1.704	0.565	9.090	1	0.003	5.497	1.816	16.645
**Received sufficient support from employer**								
Yes, absolute support			8.094	4	0.088			
Yes, some support	0.412	0.260	2.515	1	0.113	1.510	0.907	2.514
Yes, but only limited support	0.702	0.327	4.604	1	0.032	2.017	1.063	3.829
Not at all	0.403	0.434	0.859	1	0.354	1.496	0.638	3.504
Not currently employed	−0.323	0.443	0.532	1	0.466	0.724	0.304	1.725
**Received sufficient support from the Government of Canada**								
Yes, absolute support			4.089	3	0.252			
Yes, some support	0.098	0.265	0.137	1	0.711	1.103	0.656	1.857
Yes, but only limited support	0.044	0.298	0.022	1	0.883	1.045	0.583	1.873
Not at all	0.453	0.257	3.125	1	0.077	1.574	0.952	2.602
**Received counselling during the pandemic**								
No Yes	0.550	0.212	6.760	1	0.009	1.734	1.145	2.625
Constant	−0.805	1.257	0.410	1	0.522	0.447		

## Data Availability

Data for this study are available and can be released following reasonable request by writing to the corresponding author.
